# Supplementary material for: Mapping inhibitory sites on the RNA polymerase of the 1918 pandemic influenza virus using nanobodies
Source: Nat Commun. 2022 Jan 11;13:251. doi: 10.1038/s41467-021-27950-w (PMC8752864; doi:10.1038/s41467-021-27950-w)
Supplement: Supplementary file 1 — Supplementary Information File [file 41467_2021_27950_MOESM1_ESM.pdf]

Supplementary Information

Mapping inhibitory sites on the RNA polymerase of the 1918 pandemic influenza virus using nanobodies

Jeremy R. Keown[1,*], Zihan Zhu[2,*], Loïc Carrique[1,*], Haitian Fan[2,*], Alexander P. Walker[2,6], Itziar Serna Martin[2,7], Els Pardon[3,4], Jan Steyaert[3,4], Ervin Fodor[2,], and Jonathan M. Grimes[1,5,**]**

1 Division of Structural Biology, Wellcome Centre for Human Genetics, University of Oxford, Oxford OX3 7BN, United Kingdom
2 Sir William Dunn School of Pathology, University of Oxford, South Parks Road, Oxford OX1 3RE, United Kingdom
3 VIB-VUB Center for Structural Biology, VIB, Brussels, Belgium
4 Structural Biology Brussels, Vrije Universiteit Brussel, Brussels, Belgium
5 Diamond Light Source Ltd, Harwell Science & Innovation Campus, Didcot OX11 0DE, United Kingdom
6 Present address: School of Cellular and Molecular Medicine, Faculty of Life Sciences, University of Bristol, Bristol BS8 1TD, United Kingdom
7 Present address: Department of Virology, Faculty of Veterinary Sciences, Utrecht University, Utrecht, The Netherlands.
* Authors contributed equally
** Authors jointly supervised this work
[*]email: ervin.fodor@path.ox.ac.uk, jonathan@strubi.ox.ac.uk

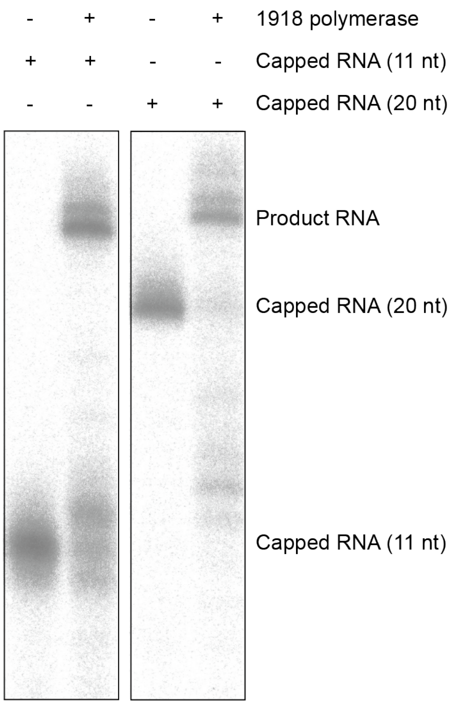

Supplementary Figure 1. Functional analysis of the 1918 polymerase. The purified recombinant 1918 polymerase is transcriptionally active in the presence of a 11 nucleotide-long capped RNA primer or a 20 nucleotide-long capped RNA requiring prior cleavage by the viral PA endonuclease. Representative gel from n = 3 independent reactions is shown. Source data are provided as a Source Data file.

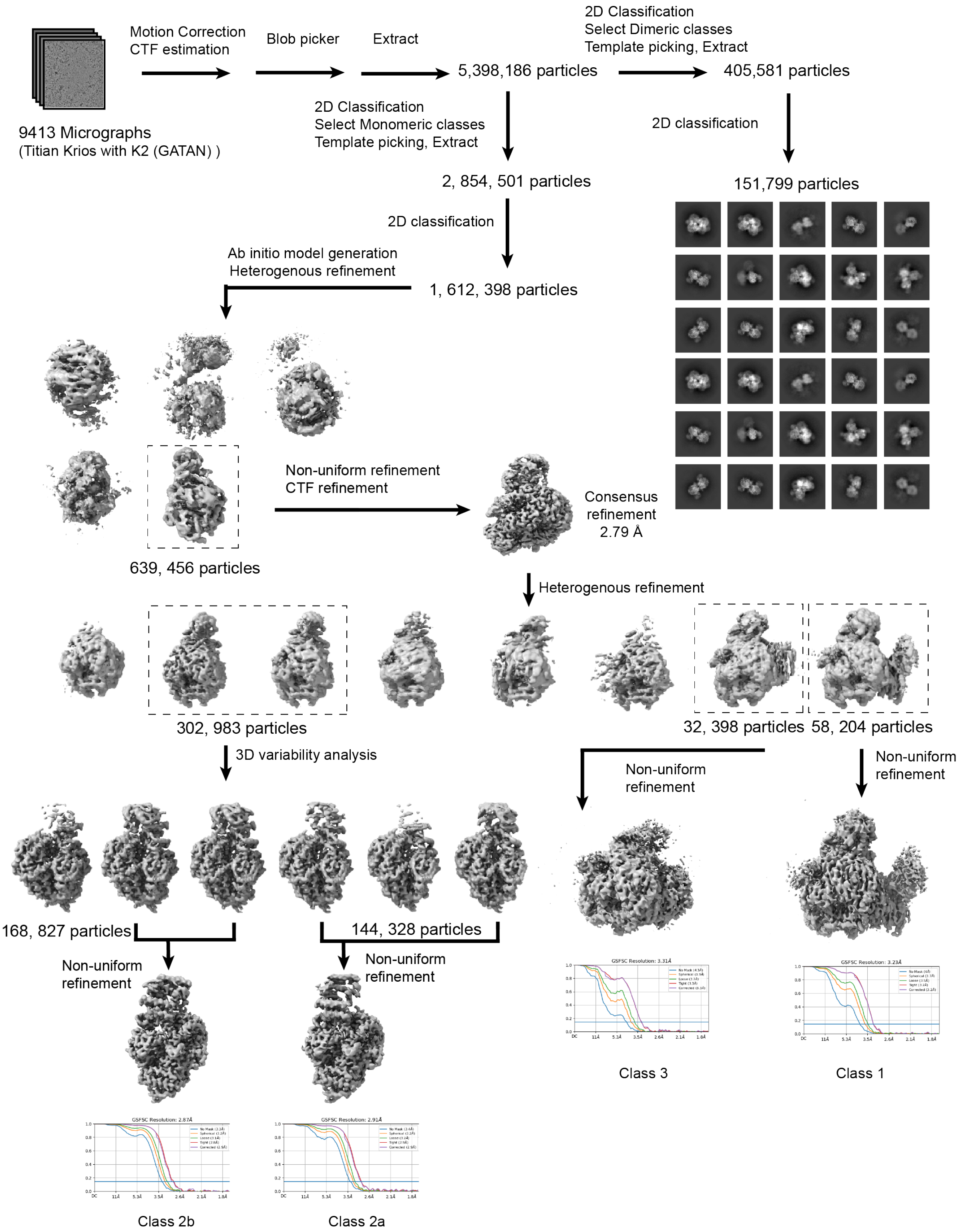

Supplementary Figure 2. Cryo-EM data processing and analysis scheme.

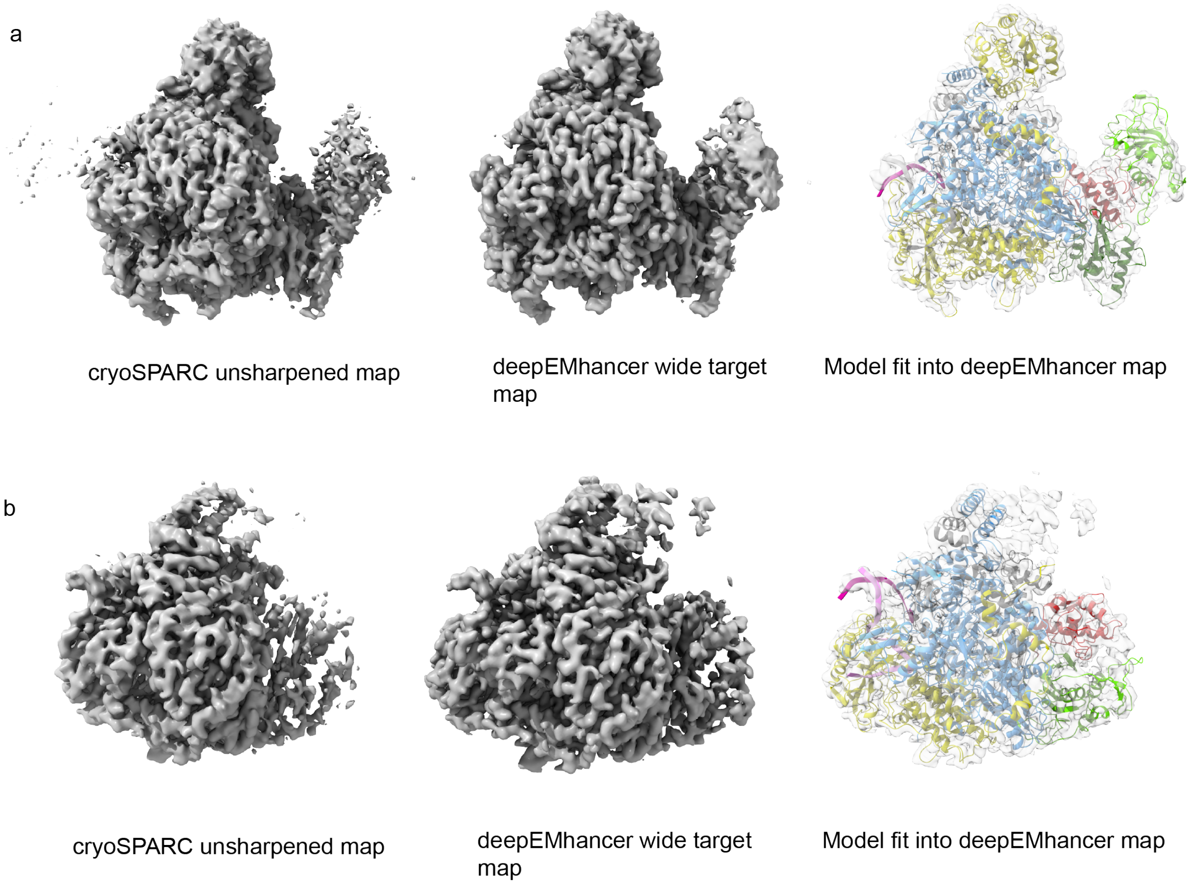

Supplementary Figure 3. Map modifications by deepEMhancer. Comparison of the cryoSPARC unsharpened and deepEMhancer processed maps of the transcriptase (**a**) and replicase (**b**) with the model fitted. Protein and nucleic acids are coloured as follows: 5′ and 3′ vRNA promoter (pink/purple), PA subunit (yellow), PB1 subunit (blue), and PB2 N-terminal region (grey), the mid-link (red), cap-binding (light green), and 627 domain (dark green).

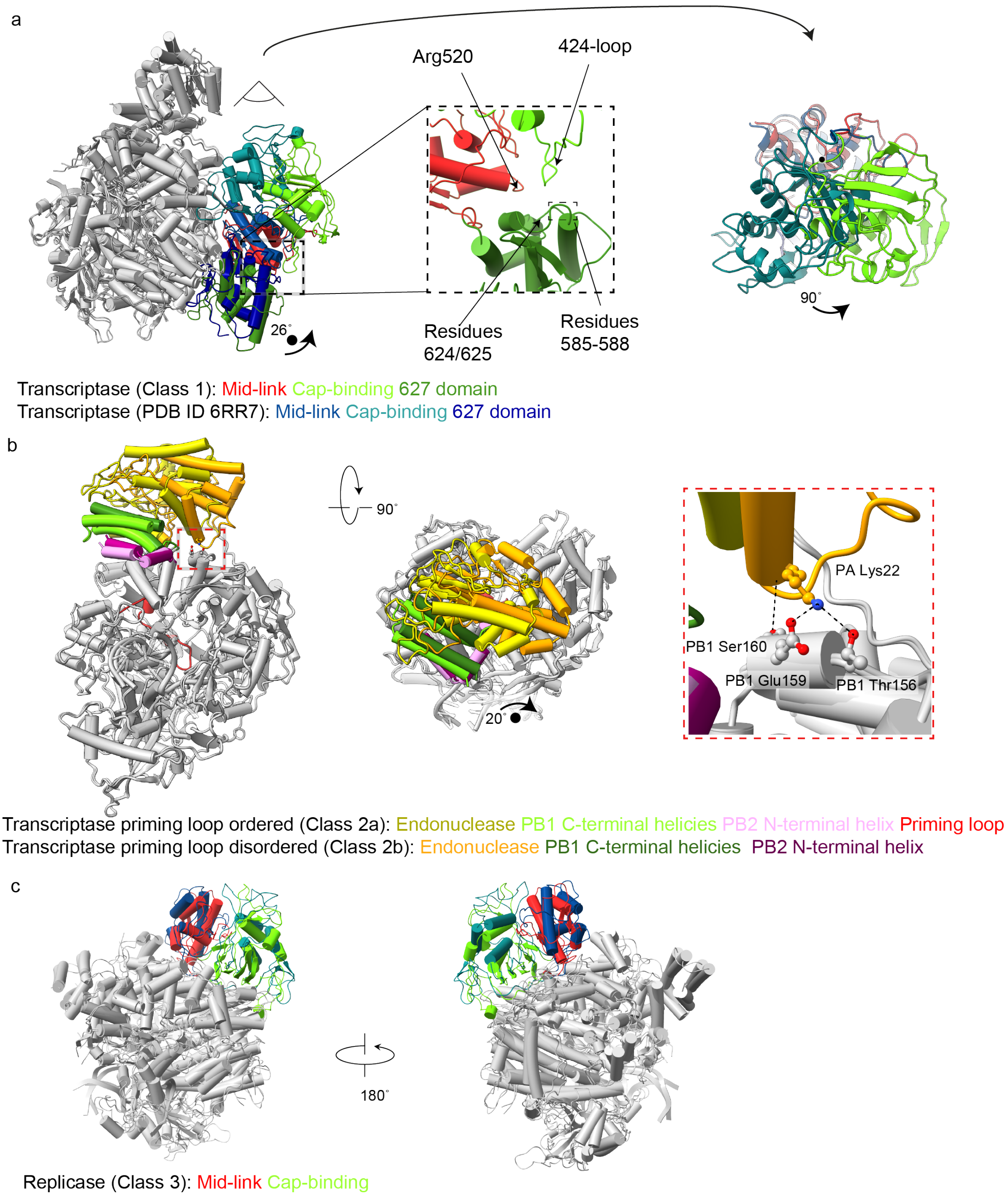

Supplementary Figure 4. Movements of the PA endonuclease and PB1 priming loop. a, Comparison of the PB2 C-terminal domains from the Class 1 conformation and transcriptase conformation (PDB ID 6RR7). Inset panel details interactions between the PB2 424-loop and the 627 domain in the Class 1 conformation. The right-hand panel demonstrates the 90º rotation of the PB2 cap-binding domain, when viewed from the top. The black dot shows the centre of rotation. **b,** Comparison of Class 2a and Class 2b conformations of the polymerase with top view showing rotation of the endonuclease by 20º between the two conformations. Inset panel details new interactions formed between PA Lys22 and residues of the PB1 core in Class 2b where the priming loop is not ordered. **c,** Comparison of the PB2 C-terminal domains between Class 3 replicase conformation and the replicase conformation (PDB ID 6XZR).

a

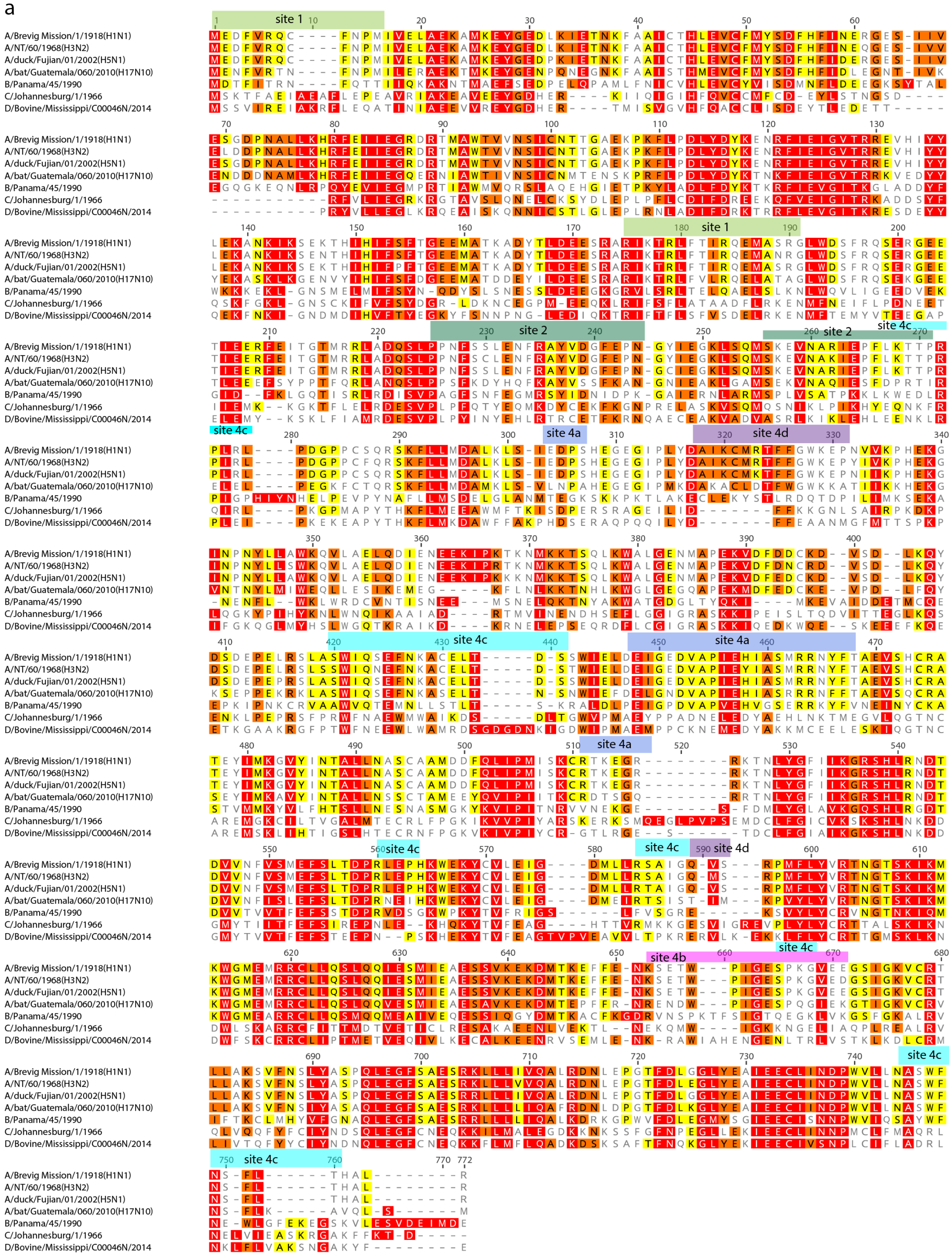
A/Brevig Mission/1/1918(H1N1)
A/NT/60/1968(H3N2)
A/duck/Fujian/01/2002(H5N1)
A/bat/Guatemala/060/2010(H17N10)
B/Panama/45/1990
C/Johannesburg/1/1966
D/Bovine/Mississippi/C00046N/2014
site 1
site 2
site 4a
site 4b
site 4c
site 4d

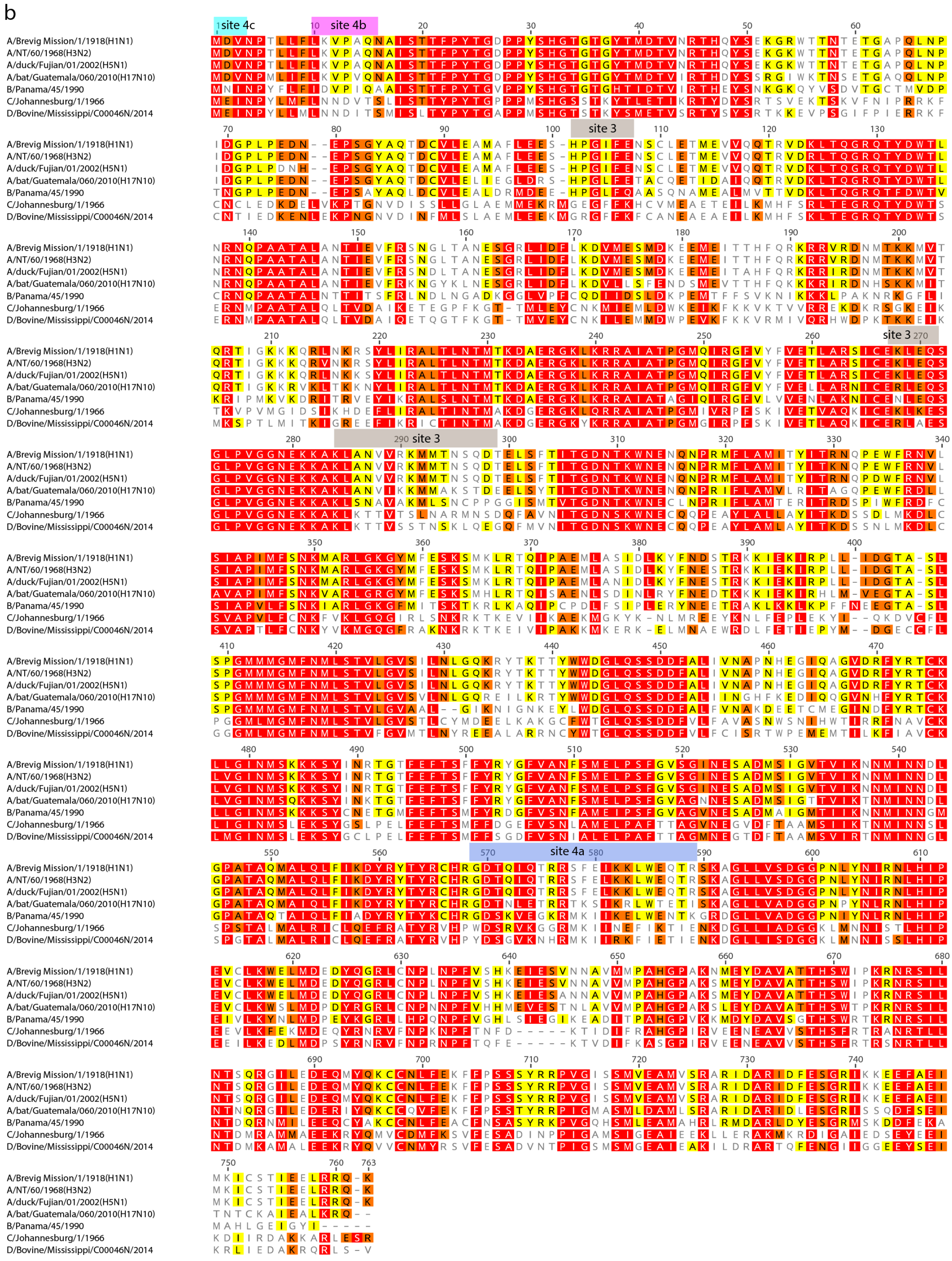
b
site 4c
site 4b
site 3
site 4a
A/Brevig Mission/1/1918(H1N1)
A/NT/60/1968(H3N2)
A/duck/Fujian/01/2002(H5N1)
A/bat/Guatemala/060/2010(H17N10)
B/Panama/45/1990
C/Johannesburg/1/1966
D/Bovine/Mississippi/C00046N/2014

c

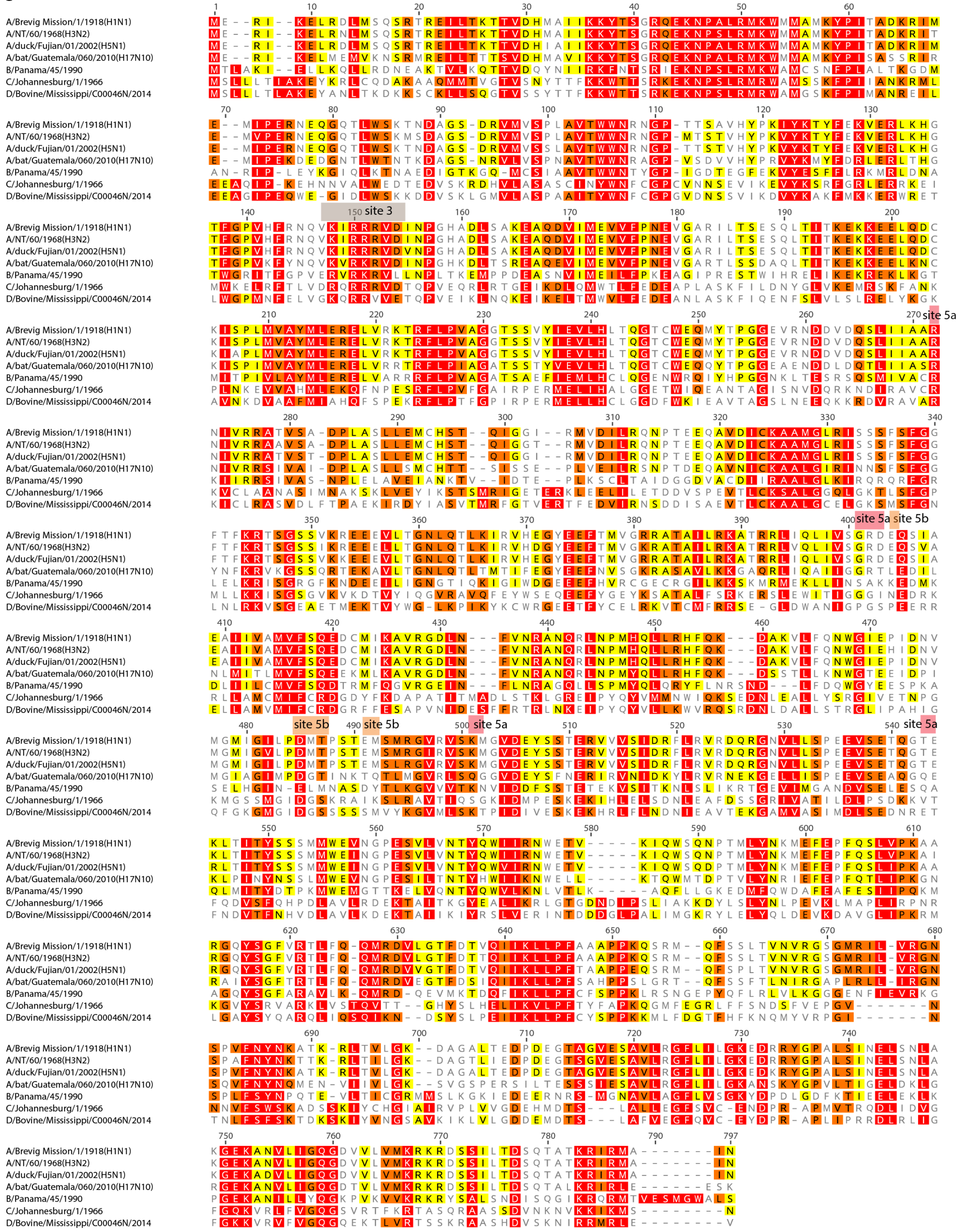

Supplementary Figure 5. Sequence similarity of influenza polymerase subunits. a-c, Sequence alignments of PA (**a**), PB1 (**b**), PB2 (**c**) from A/Brevig Mission/1/1918 (H1N1), A/NT/60/1968 (H3N2), A/duck/Fujian/01/2002 (H5N1), A/bat/Guatemala/060/2010 (H17N10), B/Panama/45/1990, C/Johannesburg/1/1966, and D/bovine/Mississippi/COOO46N/2014. Residues are coloured red, orange, yellow, white from most to least conserved. Sites of nanobody binding are annotated and colour coded. The nanobodies are grouped according to which site that bind to: site 1 (Nb8198, Nb8199, Nb8200, Nb8203, Nb8209), site 2 (Nb8202, Nb8210, Nb8204), site 3 (Nb8207), site 4a (Nb8192, Nb8205), site 4b (Nb8189, Nb8190, Nb8201), site 4c (Nb8191, Nb8196), site 4d (Nb8206), site 5a (Nb8193, Nb8194), site 5b (Nb8208).

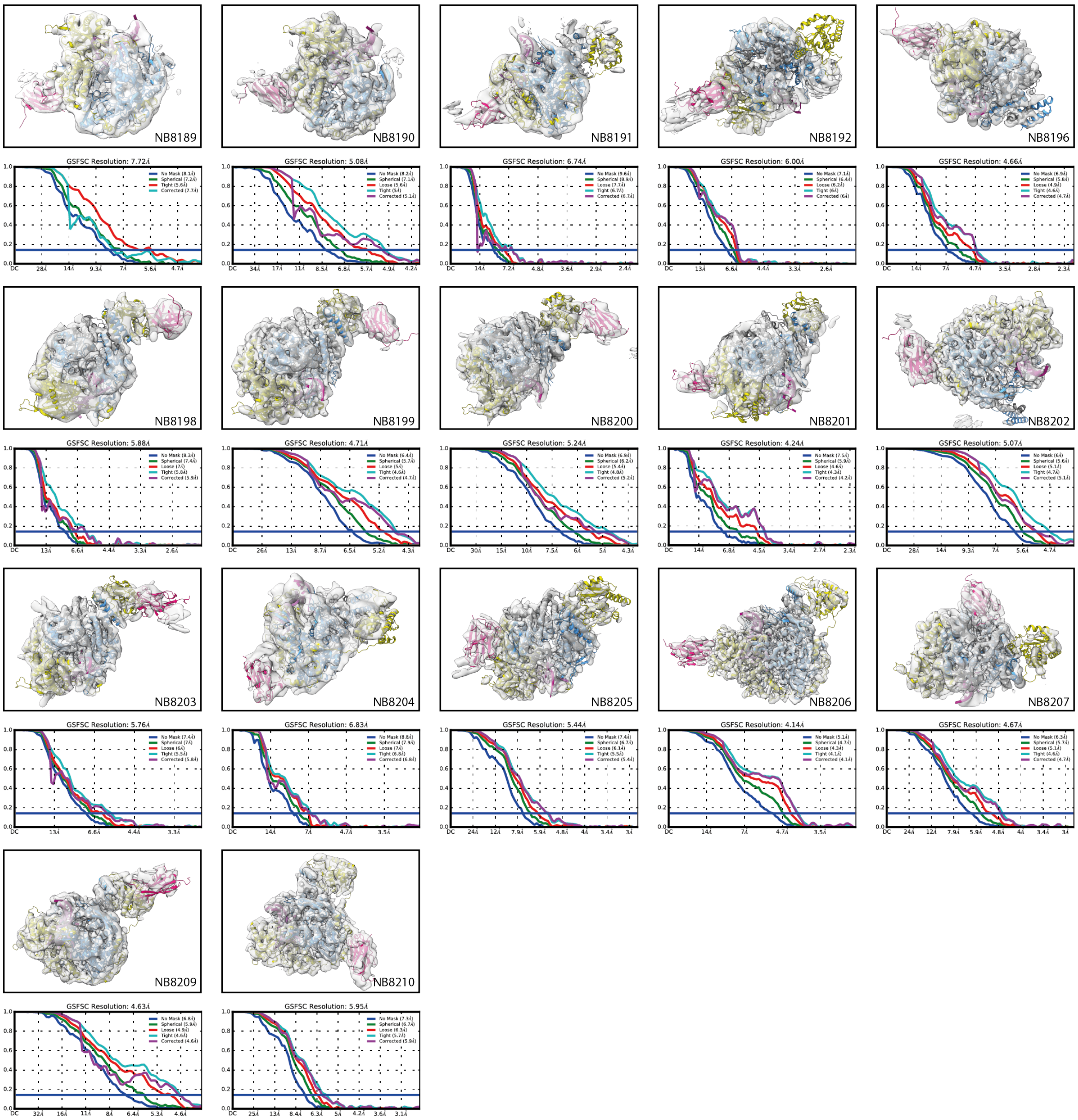

Supplementary Figure 6. Fit of the polymerase and nanobody structures into the cryo-EM maps. Protein subunits are coloured as follows: PA (yellow), PB1 (blue), PB2 (grey), 5′ and 3′ vRNA promoter (purple/pink) and nanobody (magenta). FSC plots for each map are shown.

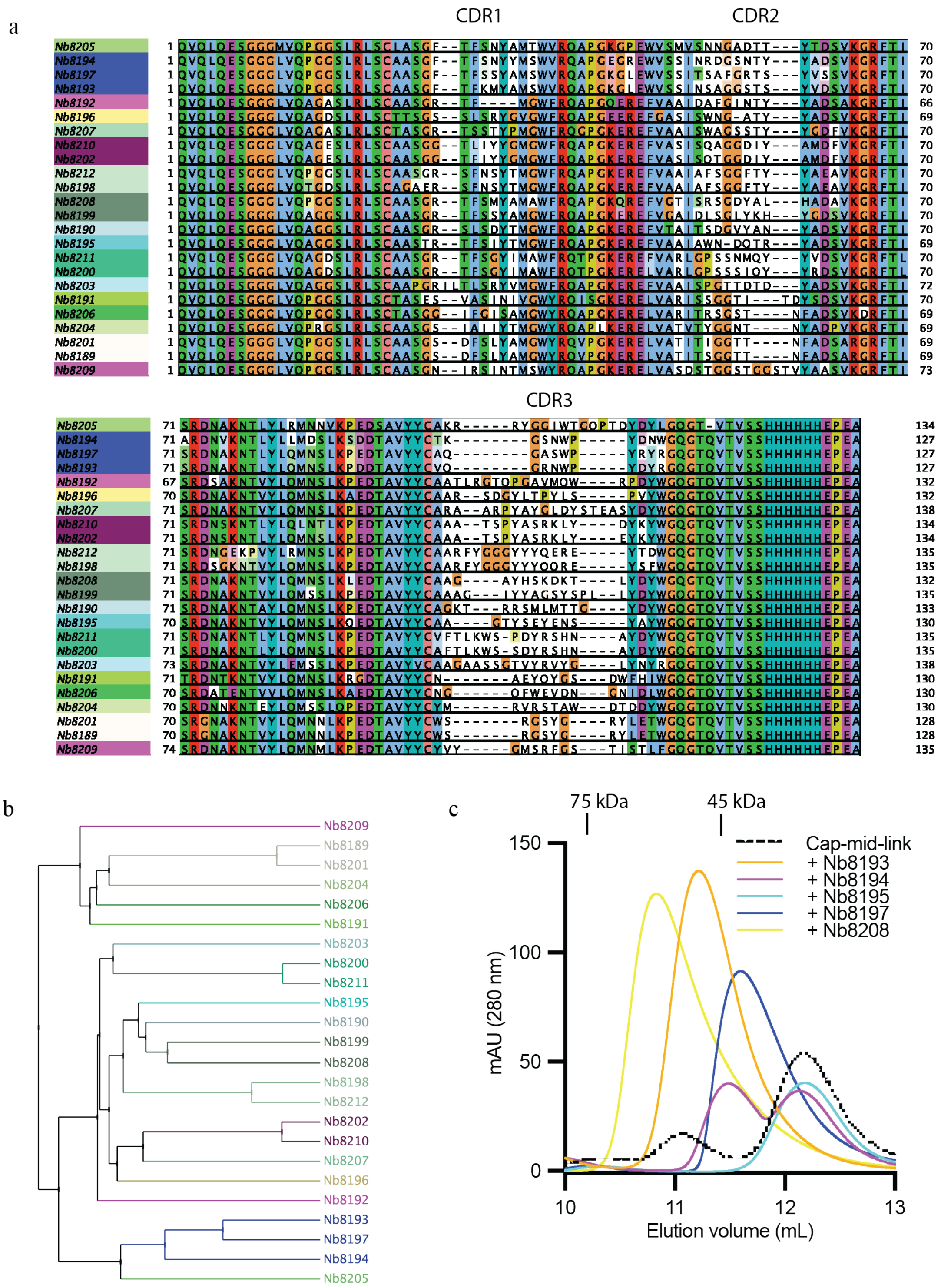

Supplementary Figure 7. Sequences of nanobodies and their relationships. a, Sequence alignment of the panel of 24 nanobodies. The approximate location of the three complementary-determining regions (CDRs) are indicated. **b,** Phylogenetic tree of the nanobody protein sequences. **c,** Size exclusion chromatograms of cap-mid-link alone or mixed with Nb8193, Nb8194, Nb8195, Nb8197, or Nb8208. Elution positions of molecular weight standards are shown.

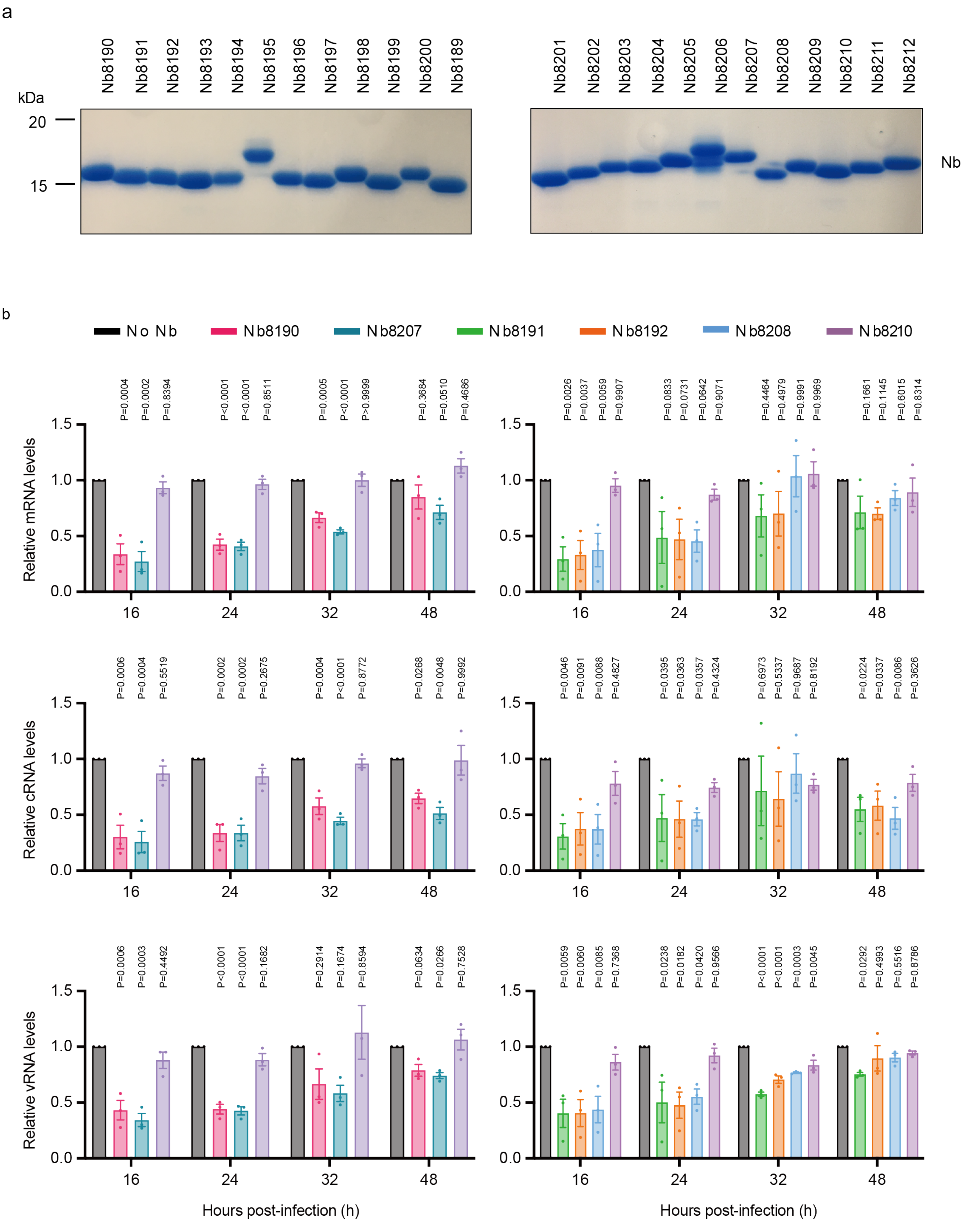

Supplementary Figure 8. Purification of nanobodies and effect of nanobodies on viral RNA levels during infection. a, Purified nanobodies analysed by SDS-PAGE and staining with Coomassie Brilliant Blue. **b,** Effect of nanobodies on mRNA, cRNA and vRNA in HEK293T cells during a course of infection with WSN-1918^{RNP} virus. Data are mean ± s.e.m. For each set of nanobodies, n = 3 independent transfections and infections. Ordinary one-way ANOVA was used for comparing mRNA, cRNA and vRNA levels separately to the control without nanobody (no Nb) at individual time points. $P < 0.05$ is considered significant. Source data are provided as a Source Data file.

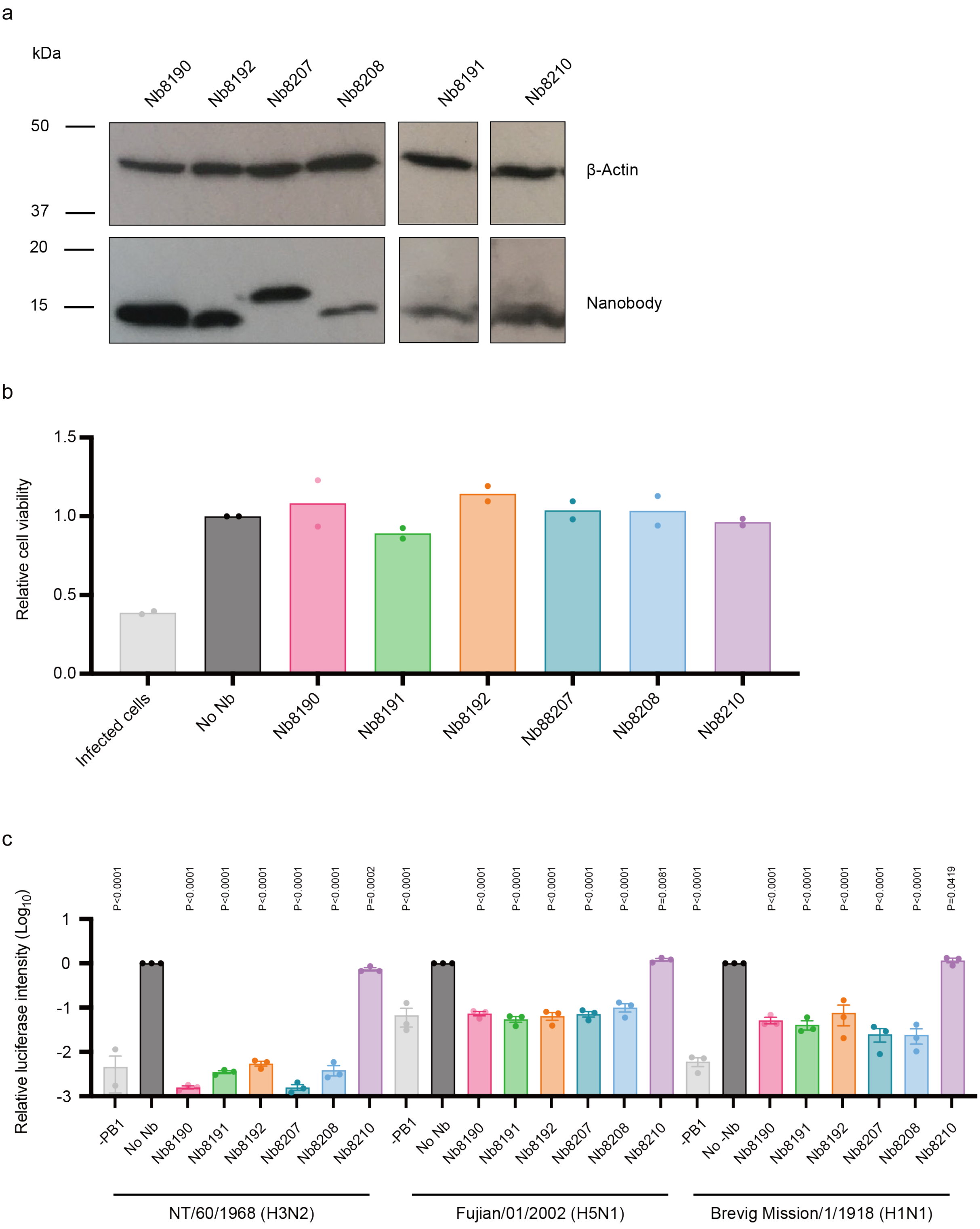

Supplementary Figure 9. Expression of nanobodies, effect of nanobodies on cell viability and nanobody inhibition across H3N2, H5N1 and H1N1 influenza A viruses. a, Expression of nanobodies in transfected HEK293T cells was analysed by SDS-PAGE and Western blotting with and anti-alpaca polyclonal antibody. β-actin was used as a loading control. A representative blot is shown. **b.** Cell viability assay of HEK293T cells transfected with plasmids to express nanobodies. Data are mean ± range. N = 2 independent transfections. **c.** Effect of nanobodies on H3N2, H5N1 and H1N1 influenza A virus polymerase activity using a luciferase reporter minireplicon assay. Data are mean ± s.e.m. n = 3 independent transfections with n = 2 technical replicates. Ordinary one-way ANOVA was used to compare the relative luciferase intensity in the presence and absence of nanobodies for each influenza A virus subtype. $P < 0.05$ is considered significant. Source data are provided as a Source Data file.

Supplementary Table. 1 CryoEM data collection and refinement statistics.

	1918_FluPol vRNA				1918_FluPol vRNA+ Nb8189	1918_FluPol vRNA+ Nb8190	1918_FluPol vRNA+ Nb8191	1918_FluPol vRNA+ Nb8192
	**Class 2a**	**Class 2b**	**Class 1**	**Class 3**				
	EMD-12322	EMD-12323	EMD-12342	EMD-12348	EMD-12361	EMD-12362	EMD-12363	EMD-12364
	PDB 7HNA	PDB 7NHC	PDB 7NHX	PDB 7NI0	PDB 7NIK	PDB 7NIL	PDB 7NIR	PDB 7NIS
**Data collection**								
Microscope	Krios (STRUBI)				Glacios (STRUBI)	Glacios (STRUBI)	Titan Krios (STRUBI)	Titan Krios (STRUBI)
Voltage (kV)	300				200	200	300	300
Detector	Gatan K2 with EF				Flacon III	Flacon III	Flacon III	Flacon III
Recording mode	Counting				Linear	Linear	Linear	Linear
Magnification	165,000				73,000	73,000	130,000	130,000
Movie/micrograph pixel size (Å)	0.825				2	2	1.1	1.1
Dose rate (e-/Å^2/sec)	12.3				32	31	34.5	34.5
Number of frames per movie	50				40	40	40	40
Movie exposure time (s)	5				2	2	1.5	1.5
Total dose (e-/Å^2)	61.5				64	62	51.8	51.8
Defocus range (um)	-1.2 to -2.5				-2 to -3.5	-2 to -3.5	-2 to -3.5	-2 to -3.5
Volta Phase Plate	no				no	no	no	no
**EM data processing**								
Number of movies/micrographs	9,413				201	187	1340	980
Box size (px)	256				140	170	260	240
Particle number (total)	2,854,501				372,000	411,000	198,000	512,000
Particle number (post 2D)	1,612,398				100,000	182,000	60,000	122,000
Particle number (post 3D)	639,456				53, 970	44, 552	33, 278	43, 976
Particle number (used in final map)	144, 328	168,827	58,204	32,398	53, 970	44, 552	33, 278	43, 976
Symmetry	C1	C1	C1	C1	C1	C1	C1	C1
Map resolution (FSC 0.143)	2.91	2.87	3.23	3.31	6.19	5.01	6.7	5.96
Local resolution range (FSC 0.5)	ND	ND	ND	ND	ND	ND	ND	ND
Map sharpening B-factor (Å^2)	-94	-91	-60	-66	-438	-321	-527	-472
**Model Building and Validation**								
Initial model used	6RR7	7HNA	6RR7/7HNA	7HNA/6QNW	7HNA and In silico generated nanobody	7HNA and In silico generated nanobody	7HNA and In silico generated nanobody	7HNA and In silico generated nanobody
Model composition								
Non-hydrogen protein atoms	28,050	27,760	34,662	29,335				
Protein residues	1,700	1680	2121	1794				
Nucleotides (RNA)	23	23	23	23				
RMSD from ideal								
Bond length (Å)	0.003	0.002	0.003	0.003				
Bond angles (°)	0.572	0.512	0.679	0.656				
Validation								
Molprobity score	1.77	1.45	1.84	1.96				
Clashscore	8.66	5.58	9.57	14.53				
Rotamers outliers (%)	0	0	0	0.06				
FSC (0.5) model-vs-map	3.2	3	3.5	3.8				
CC model-vs-map (masked)	0.83	0.82	0.81	0.75				
Ramachandran plot								
Favored (%)	95.7	97.2	95.2	95.8				
Allowed (%)	4.3	2.8	4.7	4.1				
Outliers (%)	0	0	0.1	0.1				

1918_FluPol vRNA+ Nb8196	1918_FluPol vRNA+ Nb8198	1918_FluPol vRNA+ Nb8199	1918_FluPol vRNA+ Nb8200	1918_FluPol vRNA+ Nb8201	1918_FluPol vRNA+ Nb8202
EMD-12371<br>PDB 7NJ3	EMD-12372<br>PDB 7NJ4	EMD-12373<br>PDB 7NJ5	EMD-12375<br>PDB 7NJ7	EMD-12428<br>PDB 7NK1	EMD-12429<br>PDB 7NK2
Titan Krios (STRUBI)	Titan Krios (STRUBI)	Glacios (STRUBI)	Glacios (STRUBI)	Titan Krios (STRUBI)	Glacios (STRUBI)
300	300	200	200	300	200
Flacon III	Flacon III	Flacon III	Flacon III	Gatan K2 with EF	Flacon III
Linear	Linear	Linear	Linear	Counting	Linear
130,000	130,000	73,000	73,000	130,000	73,000
1.1	1.1	2	2	1.08	2
35	35	35	35	7	35
40	40	40	40	28	40
2	2	2	2	4	2
69.9	69.9	69.5	69.5	31	69.5
-2 to -3.5	-2 to -3.5	-2 to -3.5	-2 to -3.5	-2 to -3.5	-2 to -3.5
no	no	no	no	No	no
2340	1809	1335	943	3650	863
256	240	150	150	250	140
1, 004, 000	775,000	1,874,000	1,713,000	1,790,000	1,277,000
206, 000	194,000	375,000	250,000	140,000	470, 000
93, 519	75, 917	216,550	151, 144	45, 716	198, 338
93, 519	75, 917	216,550	151, 144	45, 716	198, 338
C1	C1	C1	C1	C1	C1
4.48	5.84	4.63	4.82	4.22	4.84
ND	ND	ND	ND	ND	ND
-255	-432	-344	-351	-115	-384
7HNA and In silico generated nanobody	7HNA and In silico generated nanobody	7HNA and In silico generated nanobody	7HNA and In silico generated nanobody	7HNA and In silico generated nanobody	7HNA and In silico generated nanobody

1918_FluPol vRNA+ Nb8203	1918_FluPol vRNA+ Nb8204	1918_FluPol vRNA+ Nb8205	1918_FluPol vRNA+ Nb8206	1918_FluPol vRNA+ Nb8207	1918_FluPol vRNA+ Nb8209	1918_FluPol vRNA+ Nb8210
EMD-12430<br>PDB 7NK4	EMD-12431<br>PDB 7NK6	EMD-12433<br>PDB 7NK8	EMD-12435<br>PDB 7NKA	EMD-12437<br>PDB 7NKC	EMD-12440<br>PDB 7NKI	EMD-12447<br>PDB 7NKR
Titan Krios (STRUBI)	Titan Krios (STRUBI)	Titan Krios (STRUBI)	Titan Krios (STRUBI)	Titan Krios (STRUBI)	Glacios (STRUBI)	Titan Krios (STRUBI)
300	300	300	300	300	200	300
Flacon III	Flacon III	Flacon III	Flacon III	Flacon III	Flacon III	Flacon III
Linear	Linear	Linear	Linear	Linear	Linear	Linear
105,000	105,000	105,000	105,000	105,000	73,000	105,000
1.4	1.4	1.4	1.4	1.4	2	1.4
42.5	36.2	42.5	36.2	36.2	31.5	36.2
40	40	40	40	40	40	40
1.5	2	1.5	2	2	2	2
64.9	72.5	64.9	72.5	72.5	63	72.5
-2 to -3.5	-2 to -3.5	-2 to -3.5	-2 to -3.5	-2 to -3.5	-2 to -3.5	-2 to -3.5
no	no	no	no	no	no	no
1056	2594	340	1775	1077	1315	664
190	200	170	200	170	160	180
1,000,000	535,000	457,000	1,700,000	1,468,000	2,930,000	631,000
261,000	255,000	117,000	494,000	520,000	730,000	146,000
91,532	36,297	40,289	223,847	90,450	204,625	67,047
91,532	36,297	40,289	223,847	90,450	204,625	67,047
C1	C1	C1	C1	C1	C1	C1
5.32	6.72	5.34	4.07	4.46	4.67	5.6
ND	ND	ND	ND	ND	ND	ND
-403	-582	-394	-237	-284	-310	-451
7HNA and In silico generated nanobody	7HNA and In silico generated nanobody	7HNA and 6QPG	7HNA and In silico generated nanobody	7HNA and In silico generated nanobody	7HNA and In silico generated nanobody	7HNA and In silico generated nanobody

Supplementary Table 2. Data collection and refinement statistics.

	FJ_Capmidlink + Nb8193	FJ_Capmidlink + Nb8194	FJ_Cap-binding domain +Nb8208
**Data collection**			
Space group	P1 21 1	P 1 21 1	P 6
Cell dimensions			
a, *b*, *c* (Å)	42.58, 106.54, 98.41	55.60, 65.46, 60.0	128.20, 128.20, 70.27
α, β, γ (°)	90.0, 98.0, 90.0	90, 108.17, 90	90, 90, 120
Resolution (Å)	44.31-1.68 (1.74-1.68)	52.81-1.88 (1.95-1.88)	70.27-3.14 (3.26-3.14)
R_{merge} (ellipsoidal)	0.018 (0.92)	0.037 (1.53)	0.091 (0.50)
$I / \sigma I$	23.3 (0.57)	9.29 (0.44)	9.85 (1.1)
$CC_{1/2}$	1 (0.29)	1 (0.16)	0.99 (0.59)
Completeness (%, ellipsoidal)	70.3 (7.9)	89.92 (48.22)	92.94 (32.64)
Redundancy	2 (1.5)	2 (1.9)	2 (2)
**Refinement**			
Resolution (Å)	44.31-1.68 (1.74-1.68)	52.81-1.88 (1.95-1.88)	70.27-3.14 (3.26-3.14)
No. reflections	136,491 (1,126)	59,891 (3,437)	21,639 (754)
R_{work} / R_{free}	0.17 /0.21	0.19/0.24	0.24/0.29
No. atoms (residues)	6,671 (793)	3,247 (399)	4,437 (560)
Protein	6204	3142	4,387
Ligand/ion	37	28	50
Water	450	77	0
B-factors			
Protein	34.12	53.82	72.00
Ligand/ion	47.95	77.01	63.10
Water	38.08	49.51	0
R.m.s. deviations			
Bond lengths (Å)	0.02	0.05	0.002
Bond angles (°)	1.58	0.57	0.49
Ramachandran			
Favoured (%)	99.1	97.7	96.0
Additionally allowed (%)	0.9	2.3	3.6
Disallowed (%)	0	0	0.4

*Values in parentheses are for highest-resolution shell. * For each structure a single crystal was used.
